# Incorporating One Health into a front-line field epidemiological training programme in Papua New Guinea: lessons learned

**DOI:** 10.5365/wpsar.2024.15.1107

**Published:** 2024-11-12

**Authors:** Kelitha Malio, Bethseba Peni, Elaine Hevoho, Abel Yamba, Alois Pukienei, Laura Macfarlane-Berry, Trinidad Velasco Ortuzar, Barry Ropa, Ilagi Puana, Therese Kearns, Tambri Housen

**Affiliations:** a World Vision Papua New Guinea, Boroko, National Capital District, Papua New Guinea.; b Field Epidemiology Training Program, National Department of Health, Port Moresby, Papua New Guinea.; c West New Britain Provincial Health Authority, Kimbe, West New Britain, Papua New Guinea.; d National Agriculture Quarantine & Inspection Authority, Port Moresby, Papua New Guinea.; e Autonomous Region of Bougainville Provincial Health Authority, Buka, Autonomous Region of Bougainville, Papua New Guinea.; f University of Newcastle, Newcastle, New South Wales, Australia.

## Abstract

**Problem:**

Over the past two decades, there has been increased recognition of the importance of a more holistic approach to preventing, predicting, detecting and responding to public health threats. The COVID-19 pandemic highlighted the need to bring together environmental, human and animal health sectors in addressing public health threats and the need to develop skilled front-line workers to act as surge capacity during health emergencies.

**Context:**

Papua New Guinea is a high-risk country for emerging and re-emerging pathogens. The effects of climate change, human-mediated encroachment on natural habitats and destructive land-use practices have threatened ecosystems and caused environmental damage. The movement of goods, animals and people over porous borders provides opportunities for the introduction and spread of new pathogens.

**Action:**

In recognition of the importance of multisectoral responses to health threats in Papua New Guinea, and the need to train front-line workers, we designed and piloted a 3-month One Health in-service training programme for front-line workers from across all sectors.

**Lessons learned:**

The co-creation of curricula was essential in ensuring the relevance of the programme to front-line workers from multiple sectors, and the development of provincial training teams was key to ensuring mentorship and programme sustainability. Bringing front-line workers together in joint trainings facilitated the building of relationships, the understanding of the roles and responsibilities of the various sectors, the identification of sectoral focal points and the development of informal networks.

**Discussion:**

Papua New Guinea’s One Health front-line Field Epidemiology Training Program demonstrated that investment in cross-sectoral training programmes can be a catalyst for the implementation of One Health approaches on the front line.

## PROBLEM

Over the past two decades, there has been increased recognition of the benefits of adopting a holistic approach to preventing, detecting and responding to public health threats, one that acknowledges the interconnection of human, animal and environmental health. (*1*-*5*) While human health, animal health and environmental sectors have effectively worked together to respond to specific health emergencies, such as natural disasters, zoonotic influenzas and the Zika outbreak, (*4*, *6*) in more recent years, the importance of developing ongoing intersectoral working relationships has become increasingly apparent. The COVID-19 pandemic highlighted the need for greater mutual understanding across sectors to facilitate the collaboration needed to develop a surge workforce with the capacity to respond to health emergencies. Joint capacity-building (*2*, *7*, *8*) has emerged as an important component of strategies to foster ongoing working relationships between sectors involved in mounting national responses to public health threats.

Front-line field epidemiology training programmes (f-FETPs) are 3-month competency-based in-service training programmes that build capacity in disease surveillance, outbreak investigation, data analysis and interpretation, as well as communication to support evidence-based decision-making. Such programmes are typically offered as the first tier of a cascaded field epidemiology training model that includes a 9-month intermediate programme and a 2-year advanced programme. (*9*)

As Global Outbreak Alert and Response Network (GOARN) partners, the Field Epidemiology Training Program of Papua New Guinea (FETPNG) and the University of Newcastle, Australia, are committed to strengthening national and subnational capability to prevent, predict, detect and respond to health alerts. Recognizing the urgency of developing a multisectoral front-line workforce capable of addressing public health threats, FETPNG collaborated with the University of Newcastle to develop and pilot a One Health training programme tailored to the needs of front-line workers in Papua New Guinea.

## CONTEXT

Located in the South Pacific, Papua New Guinea is intrinsically linked to Asia, the Pacific and the rest of the world by air, sea and a land border with Indonesia. The country has widespread trade and travel with its neighbours, with the movement of goods, people, plants and animals placing it at high risk for the introduction of high-impact diseases and disease emergence. (*6*, *10*)

Papua New Guinea faces serious health threats including vaccine-preventable disease outbreaks, zoonotic disease outbreaks, neglected tropical diseases, the expansion of vector-borne diseases, antimicrobial resistance, environmental contamination, transboundary animal disease incursions, natural disasters and food security, among others. (*6*, *10*) While specific One Health activities have been implemented, such as the adoption of a national approach for combating antimicrobial resistance, (*10*) there is a lack of training programmes that integrate One Health principles.

In contrast with global trends towards increasing urbanization, less than 14% of Papua New Guinea’s population lives in urban centres. (*11*) For much of the country’s rural population, front-line workers are most likely to be the primary contact in the event of a health emergency, providing the bridge between local communities and the various sectors responsible for protecting public health. This is even more likely in the more remote, less accessible areas, where front-line workers are often the first to hear of health threats affecting communities. The importance of training front-line workers in prevention, prediction, detection and response to health threats is, therefore, especially urgent in countries like Papua New Guinea. Coordinated training of cross-sectoral teams provides opportunities for building relationships, sensitizing participants to the respective roles and responsibilities of various sectors and enhancing capacity in information sharing, collaboration and coordinated joint responses.

In 2013, led by the National Department of Health (NDoH), the FETPNG was launched with an intermediate-level programme targeting public health professionals (Field Epidemiology Training Program of Papua New Guinea. Our workforce, our future: strategic plan 2023–2028. Internal document). However, no equivalent programme was offered to those working in the animal health and environmental sectors. Recognizing the need for a multisectoral training programme to strengthen field epidemiology capability on the front line, the One Health f-FETPNG was initiated. The primary focus of f-FETPNG is to equip front-line workers across sectors with the necessary competencies to prevent, predict, detect and respond to public health threats. Additionally, f-FETPNG aims to foster stronger communication with communities and collaboration across public health, animal health, plant health and conservation sectors at the provincial level. This report describes the methods employed in developing and implementing this training programme in four provinces and lessons learned along the way.

## ACTION

In September 2022, the NDoH received a grant from the Global Fund COVID-19 Response Mechanism (*12*) to pilot a 3-month in-service FETP. An initial stakeholder consultation determined the principles and main components of the training programme, which were further refined during programme implementation in an iterative process. The elements covered included the development of pilot site selection criteria, provincial assessment visits, curriculum development, training of trainers (ToTs) in adult principles of learning, development of participant selection criteria and quality assurance processes. The decision to adopt a One Health approach was taken early on and played a major role in shaping the format and content of the f-FETPNG pilot. The primary aim was to strengthen disease surveillance, early detection and response across sectors. Emphasis was placed on collaboration and communication skills to facilitate teamwork across disciplines and sectors. Field projects were designed to give participants practical experience in applying epidemiological principles, while the face-to-face workshops provided an opportunity to build relationships across sectors, sensitize participants to One Health principles and expose them to other sectors’ operations.

**Fig. 1:**
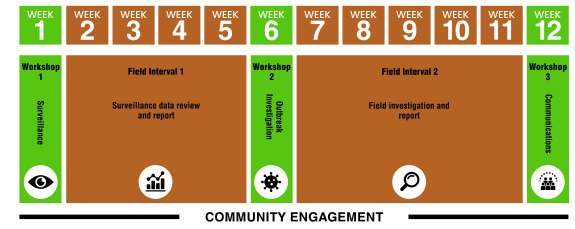
Phases of the One Health front-line Field Epidemiology Training Program of Papua New Guinea, 2023

In contrast to the national intermediate-level FETPNG, the f-FETPNG was designed to be administered at provincial level. Thus, the building of provincial capacity to successfully roll out and replicate training for subsequent cohorts of front-line workers was the main aim of the pilot training programmes. The initial selection of pilot provinces was governed by several factors, including: (i) the presence of intermediate FETPNG graduates who could serve as trainers and mentors and deliver the training programme in collaboration with identified trainers and mentors from other sectors; (ii) the presence of a strong provincial health authority (PHA); and (iii) the prioritization by the National Agriculture Quarantine and Inspection Authority (NAQIA). Five pilot provinces were selected using these criteria: Eastern Highlands, Morobe, National Capital District, West New Britain and West Sepik. The 3-month in-service training programme was delivered in each province to a single cohort between 2022 and 2024. This enabled the training materials to be reviewed and adapted after each cohort, creating an iterative improvement process. The f-FETPNG training programmes consisted of 3-week face-to-face workshops, 1 month apart. In the intervals between workshops, fellows were expected to carry out two field projects, comprising a surveillance task and a field or outbreak investigation (**Fig. 1**). For this part of the training, fellows were required to either collect their own data or use existing data from their workplace. They were supported by faculty members who provided guidance on collecting and analysing data.

[**insert** Figure 1]

As One Health front-line field epidemiology curricula were unavailable during the programme development phase, existing sector-specific resources were adapted, including the United States Centers for Disease Control and Prevention’s *FETP-frontline curriculum guide*, (*13*) the Frontline In-Service Applied Veterinary Epidemiology Training (ISAVET) programme (*14*) and the intermediate FETPNG. Core competencies focused on epidemiological principles using a One Health approach, surveillance and data analysis, outbreak/field investigation and community engagement (**Table 1**). As part of the community engagement component, f-FETPNG programme fellows were tasked with identifying community priorities and developing and implementing engagement and communication plans for field projects. The aim was to strengthen relationships and trust between front-line workers and the communities they serve.

**Table 1:** Core competencies of the One Health front-line Field Epidemiology Training Program of  Papua New Guinea, 2023

Competency domain	Competency
**Epidemiological concepts**	**Describe key disease prevention and control concepts using a One Health approach**
**Epidemiological surveillance**	**Map a surveillance system for a human, animal, plant or environmental health issue** **Summarize, analyse and interpret human, animal, plant and environmental health ** ** surveillance data**
**Outbreak/field investigation**	**Investigate a public/animal/plant/environmental health alert**
**Data management and analysis**	**Create and manage epidemiological data** **Conduct a descriptive data analysis of time, place and person/animal/plant**
**Evidence-based practice**	**Provide recommendations from field projects**
**Communication and community engagement**	**Create a communication plan for sharing surveillance data and for use during an ** ** outbreak investigation** **Demonstrate capability in successfully partnering with communities**

Initially, trainers and mentors were selected from various organizations such as the NDoH, PHAs, NAQIA, the Department of Agriculture and Livestock, the Conservation Environment Protection Authority and other partners. Subsequently, graduates of the f-FETPNG were appointed as junior trainers and mentors. Additionally, faculty members included individuals who had completed the national intermediate FETP and advanced FETP, as well as those from NAQIA who had finished the Asia Pacific Consortium of Veterinary Epidemiology training programme. A ToT workshop was conducted after the third f-FETPNG pilot to provide junior faculty members with training in adult learning principles, session plan development and interactive experiential learning techniques.

Each sector was responsible for selecting its trainees for the programme. Fellow selection was based on specific criteria, including being employed in a role where they could apply their skills, having support to complete field projects during work time and being able to attend all three face-to-face workshops. The f-FETPNG fellows were matched with mentors based on sector and area of expertise. The number of fellows per cohort was limited to 20 to ensure an average of two or three mentees per mentor. A team mentoring approach was adopted to ensure less experienced mentors received support and could learn from those with more mentoring experience.

Continuous quality assurance was built into the training programme with pre-workshop facilitator sessions, participant feedback during workshops, peer feedback for trainers, workshop evaluations and a post-pilot curriculum review workshop. For instance, before the delivery of each face-to-face workshop, faculty met over 4–5 days to review content, share sectoral experiences, develop session plans and practise delivering content. During the interactive workshops, fellows provided daily feedback, which was used to adapt training content and delivery as needed. Following each workshop, a half-day post-training faculty evaluation workshop was held to reflect on four key areas: session planning, teaching, learning and mentoring. Evaluation findings informed the delivery of subsequent workshops. When the training was completed in the first four pilot provinces, a curriculum review workshop was held with representation from all sectors, faculty and graduates. The curriculum was reviewed and updated to ensure sectoral relevance. The revised curriculum was implemented in the fifth pilot province, Eastern Highlands.

While identifying suitable case studies and examples from plant health, wildlife and conservation sectors was an initial challenge, the adoption of an iterative co-creation approach enabled participants and faculty from all sectors throughout the pilot process to shape and adapt the curricula to local needs. The fellows’ engagement with trainers and mentors from different sectors during the training provided an opportunity for them to learn more about how each sector operates, their data collection and reporting pathways, and where field epidemiology skills could be used to strengthen systems in each sector. The above-mentioned daily evaluations and the end-of-workshop evaluations provided valuable examples and guidance for sourcing environmental case studies and examples in the plant health and conservation sectors. The fellows’ information on their own projects added to the pool of resources to be used when creating case studies for the course curricula.

## LESSONS LEARNED

As of October 2024, of the 99 fellows recruited from the five pilot provinces to attend f-FETPNG, 75 had successfully graduated from the programme. They included community health workers, health extension officers, rural development officers, laboratory assistants, tree and food crop officers, fisheries officers, agriculture quarantine officers, animal health officers, extension officers, environmental health officers, livestock officers, defence force medical officers, customs officers, environment and conservation officers, ports officers, public health surveillance officers and health promotion officers. A total of 150 projects were undertaken by the f-FETPNG graduates while taking part in the 3-month training programme, with all 75 graduates completing their two required projects. Surveillance projects covered topics as diverse as malaria, food handling, water quality, coconut rhinoceros beetles, livestock management and waterborne diseases after natural disasters. Outbreak/field investigation projects included yaws, beef cattle diarrhoea, diarrhoea in displaced populations after natural disasters, fish kills, pig deaths, locust plague, sugarcane diseases and several vaccine-preventable diseases.

The implementation of the One Health f-FETPNG has provided valuable insights and lessons in addressing the complex health challenges faced by the country. Key lessons are summarized below.

### Lesson 1: Tailor the programme to the local context

When developing training programmes aimed at front-line workers, it is essential to gather input from the fellows to ensure relevance to their role and sector. The co-creation approach ensured that the programme's curricula were tailored to address the specific needs and realities they faced across sectors in Papua New Guinea. Embedding continuous quality assurance from the outset facilitated continual reflection and adaptation of the programme to suit learner needs. A one-size-fits-all approach is unlikely to meet the needs of front-line workers. It is, thus, recommended that curricula development be an ongoing iterative process, that is, during not only the pilot phase but also subsequent phases.

### Lesson 2: Front-line worker relationships are important in ensuring the success of a One Health approach

As the global community shifts towards the integration of human, animal and environmental health, the need to build cross-sectoral relationships and trust has taken centre stage. The cross-sectoral training of front-line workers by the f-FETPNG has led to the formation of active informal networks and communication channels between participants through social media platforms such as WhatsApp. After the completion of the training programme, graduates continued to utilize this network to share important alerts and information, such as dead whale sightings, contaminated fish sales, community diarrhoeal deaths, foodborne illnesses and suspected disease outbreaks, as well as other health issues and concerns. These shared alerts have prompted multisectoral investigations in various locations, showcasing the lasting positive impact of the training programme on collaboration and information sharing among participants. In addition to sharing alerts, graduates have used social media to seek advice from peers on topics ranging from a dog with ocular growths, a broken sewage pipe, the environmental health impacts of a volcano and laboratory results follow-up, to shared outbreak reports and policy documents. Cross-sectoral training has enabled cross-learning, the recognition of fellows’ roles and responsibilities and the establishment of trusted relationships, which in turn have fostered collaboration, information sharing and effective multisectoral responses.

### Lesson 3: Training of trainers has a translational impact on programme delivery

ToT in the principles of adult learning led to greater ownership of the delivery of workshop sessions and high levels of participant satisfaction. Training in interactive methods helped trainers increase their confidence and ability to prepare and deliver experiential adult learning. Pre-workshop days enabled trainers to review workshop content and incorporate earlier evaluation feedback and province-specific examples, build confidence in developing session plans and provide opportunities to practise a variety of experiential learning techniques. Peer review ensured the continued growth and development of trainers.

### Lesson 4: Build a provincial training model for front-line workers

The f-FETPNG pilot has underscored the benefits of provincial-level programme implementation, enabling relationships to be forged between individuals who would possibly work together after graduation. The impact of this approach, highlighted in Lesson 2, may not have been as strong if the programme had drawn participants from across different provinces. Training provincial faculty has also equipped individual provinces with the capability to deliver similar trainings to future cohorts of front-line workers.

These lessons learned from the f-FETPNG are likely to be applicable to other island nations. The programme’s design and approach, emphasizing the importance of a coordinated and interdisciplinary approach to addressing evolving health challenges, can serve as a valuable model for countries seeking to enhance their epidemiological capacity at subnational level. Similarly, with its focus on equipping front-line workers with foundational competencies, the f-FETPNG model provides a tool that strengthens national capacity to prevent, predict, detect and control health threats, and also produces evidence for decision-making, improves communication with communities and fosters collaboration across sectors.

## Discussion

Globally, the intersection between human, animal and environmental health and the need for holistic solutions in addressing new and emerging health challenges is increasingly recognized. (*2*, *15*) Now, perhaps more than ever, front-line workers form the foundation of a nation’s health security architecture. The f-FETPNG has demonstrated that bringing front-line workers from multiple sectors together can forge strong cross-sectoral relationships that continue to have a translational impact after programme completion. Joint trainings enable a better understanding of sectoral roles and responsibilities, build trusted relationships, and increase knowledge of specific health threats facing each sector and those that are interconnected across sectors. The successful implementation of the f-FETPNG has yielded important insights into how to address complex health challenges more effectively through combined efforts. By adopting a customized, collaborative One Health approach, the programme has effectively enhanced the capabilities of front-line workers to jointly tackle health issues in Papua New Guinea.

In Papua New Guinea, where over 85% of people live in rural and/or remote locations, front-line workers are critical in identifying and responding to health threats. Multisectoral training has built foundational epidemiological capacity and has provided front-line workers with a common language and understanding. In addition, the relationships and trust formed during the training have translated into enhanced communication and collaboration through active informal networks and joint responses to alerts after completion of the programme.

The f-FETPNG will continue to evolve as lessons are identified and the programme is rolled out in different provinces, each of which will have its own unique requirements. We acknowledge the need for sustained engagement across all sectors, particularly since the programme is embedded within national and provincial health authorities. We also recognize the need to ensure graduates are engaged in activities and professional development opportunities that build upon the competencies gained during their f-FETPNG participation. We hope the lessons shared here can serve as a guide for other nations initiating One Health in-service training for front-line workers.
